# “This Group is Like a Home to Me:” understandings of health of LGBTQ refugees in a Swedish health-related integration intervention: a qualitative study

**DOI:** 10.1186/s12889-022-13641-8

**Published:** 2022-06-23

**Authors:** Pelle Pelters, Krister Hertting, Catrine Kostenius, Eva-Carin Lindgren

**Affiliations:** 1 School of Health and Welfare, Halmstad University, Halmstad, Sweden; 2 Department of Education, University of Stockholm, Stockholm, Sweden; 3 Department of Health, Education and Technology, Luleå University of Technology, Luleå, Sweden

**Keywords:** Civil society, Intervention, Integration, LGBTQ refugees, Wellbeing, Understandings of health, Qualitative content analysis

## Abstract

**Background:**

When large numbers of asylum seekers immigrate to a country, civil society is encouraged to contribute to their integration. A subgroup of asylum seekers comprising lesbian, gay, bisexual, transgender, or queer (LGBTQ) refugees are specifically deemed vulnerable to developing health and integration problems due to the double stigma of being a sexual/gender minority and a refugee. The Swedish Federation for LGBTQ Rights (RFSL) is a civil societal organization that has established the support group “RFSL Newcomers,” a health-related integration intervention that targets such refugees. The aim of the present study is reconstructing the subjective understanding of health of LGBTQ refugees.

**Methods:**

Eleven participants in Newcomers and eight organizers were interviewed about LGBTQ refugees’ experiences of migrating and participating in RFSL Newcomers. Qualitative content analysis was used to reconstruct subjective understandings of health that were constructed in these narratives. As the data did not originally concentrate on exploring understandings of health, a broad theoretical approach was used as a heuristic for the analysis, which focused on the common everyday approach of conceptualizing health as wellbeing.

**Results:**

The narratives revealed three interconnected, interdependent categories of understanding health in which tensions occur between wellbeing and ill-being: belonging versus alienation, security and safety versus insecurity, and recognition versus denial. The categories contribute to an overarching theme of health as framed freedom – i.e., freedom framed by conditions of society.

**Conclusions:**

For our participants, belonging, recognition, and security/safety are conceptual elements of understanding health, not its social determinants. Thus, these understandings emphasize relational and existential meanings of health (theoretical implication). As for practical implications, the understandings of health were connected to being either inside or outside the Newcomers group and a new society, depending on whether LGBTQ refugees comply with social requirements. As a significant actor that is representative of the cultural majority and a facilitator of LGBTQ refugees’ resettlement process, RFSL provides LGBTQ refugees with crucial orientations for becoming a “good migrant” and a “good LGBTQ person,” yet a “bad bio-citizen.” Generally, organizers of interventions may enhance the effectiveness of their interventions when relational, existential, and biomedical understandings of health are all incorporated.

## Introduction

Migration has been a natural part of a globalized world, and more than 250 million people worldwide have migrated to achieve economic, political, and social objectives. Globally, 70.8 million people have been forcibly displaced from their homes due to conflicts and climate change [1]. In 2015, 156,000 asylum seekers migrated to Sweden [1], which was the fourth-largest recipient of asylum applications in the EU in 2017 [2]. The influx of migrants into Sweden poses challenges to Swedish society in receiving and guiding refugees in the resettlement process.

Civil society is highlighted as an important co-actor in the integration of newcomers [3–5] and can be essential to channeling social capital and benefits to economically and socially disadvantaged groups [6–8]. When states increase restrictions on immigration, civil society intervenes to play a crucial role in meeting refugees and other immigrants facing challenging situations. While civil society organizations can function as advocates for newcomers, they rarely challenge central notions of government policy [4]. The Swedish government has encouraged civil society organizations, such as The Swedish Federation for Lesbian, Gay, Bisexual, Transgender and Queer Rights (RFSL), to play a supportive role in the resettling process and developing sustainable ways of integrating people into Swedish society [9].

Among migrants, lesbian, gay, bisexual, transgender, or queer (LGBTQ) refugees and other migrants (refugees hereafter) represent a subgroup whose number is hard to determine as the Swedish Migration Board does not publicize relevant statistics [10]. Nevertheless, LGBTQ refugees are at particular risk of being distressed and have unique social needs to overcome immigration stressors and social isolation and to gain understanding of the legal system [11, 12], which may lead to higher suicide risks and other detrimental mental health outcomes. Some studies posit that worse sexual health outcomes and lower beneficial behaviors occur among LGBTQ people in general. These affect the physical health of native LGBTQ people and hence LGBTQ refugees as well [13, 14]. Moreover, LGBTQ refugees struggle to secure residence in the new homeland and meet expectations of being grateful regarding their migration to a “liberation nation” [15], which adds to the stigma and minority stress experienced by native LGBTQ people [12]. Thus, LGBTQ refugees are potentially affected by two intersecting forms of structural stigma in their receiving countries: one directed towards sexual or gender minorities, and the other toward being a refugee/migrant [16–18]. Integration in the new home country is especially challenging to achieve as it is characterized by experiences of dependence, precariousness, and a continued lack of freedom [19].

Due to this double jeopardy, the health of LGBTQ refugees is generally considered a critical research interest. However, one barrier concerning current and future research may be the common reference to the biomedical health discourse, which defines how health is understood and framed. That presented “truth” about health might not match refugees’ understandings of health, which may impede the development of functional health-related interventions. However, there are few investigations on health understandings and discourses among migrants and hence LGBTQ refugees as far as we know. To fill this research gap, the aim of this explorative study is to reconstruct the understandings of health of LGBTQ refugees who participate in a Swedish health-related integration intervention group, “RFSL Newcomers,” and RFSL officials organizing the intervention.

## Materials and methods

### Setting

The study was conducted within the intervention “RFSL Newcomers,” which is run by RFSL, a Swedish non-profit and democratic organization founded in 1950. The federation aims to achieve the same rights, possibilities, and obligations for LGBTQ people as the rights enjoyed by non-LGBTQ people. RFSL is a Swedish civil society organization that has approximately 7,000 voluntary members [9]. For a long time, RFSL has been a strong advocate for LGBTQ rights and can be considered as an independent organization that carries the voices of LGBTQ people in the political context. In 2014, “RFSL Newcomers” was initiated at RFSL as a group for LGBTQ migrants (undocumented and otherwise), refugees, asylum seekers, newcomers, etc., with the aim of strengthening the voices, identities, and capacities of participants within local group meetings. Moreover, RFSL aims to support refugees who flee persecution in their home countries due to sexual orientation, gender identity, or gender expression, as well as helping them to share experiences and enjoy extended social networks [20]. RFSL also aims to offer this support due to their own experience [11].

The RFSL Newcomers groups are health-related integration interventions that address issues of both health and integration. Presently, a total of 16 active local Newcomers groups are registered in Sweden. The study was conducted in three of the groups’ locations in the northern, eastern, and western parts of Sweden, which have different sizes and levels of support activities. Due to the variety of LGBTQ target groups in RFSL Newcomers, we could use different designations for their participants. However, the term “LGBTQ refugee” is preferentially used as it accurately describes most of the study participants. Furthermore, the word “Newcomers” with a capital N refers to the group, not individual members.

### Theoretical framework

This study is part of a larger explorative study (which has been reported elsewhere; *a reference will be added after acceptance*) that investigates the experiences of LGBTQ refugees during their migration process with focus on the role of the Newcomers groups, LGBTQ refugees’ thoughts about the future, and health’s implicit role. Due to this context, LGBTQ refugees’ understandings of health have been explored not by explicitly discussing health-related definitions and practices in the interviews, but by extracting information implicitly related to the refugees’ understandings of health from interview data on the migration process.

To achieve this goal, we apply a broad health concept as a heuristic to help structure and guide our attention in the analysis. We base our conceptual approach on Kickbusch’s notion of health [21]. She describes health as a comprehensive category in so-called health societies – i.e., societies characterized by health (as risk societies are considered characterized by risk). According to this view, health penetrates all areas and aspects of life, such as individual, political, social, and economic fields and institutions. From this point of view, theoretically, every aspect of life appears to be potentially health related. In other words, health is potentially everywhere and included in all decisions regarding people’s (well-)being. On the other hand, we conceptualize health as congruent to subjective wellbeing [22], which implies the consideration of health as the same as subjective wellbeing. This understanding is commonly used in everyday life [23, 24] and is therefore deemed suitable for this study. This equating of health and wellbeing comprises the usual tension between “good” and “bad” versions of health as a tension between wellbeing and ill-being, hence emphasizing a subjective point of view [25]. Data were analyzed based on the assumption that all narrated influences on subjective experiences of wellbeing and ill-being are relevant and significant to subjective understandings of health.

The public health perspective of our study can first be derived from the World Health Organization’s claim that there is no public health without refugee and migrant health [26]. Further, we adhere to a public health perspective in terms of “utilising [subjectively experienced] wellbeing as a driver for cross-cutting public health in challenging economic and organisational contexts, acknowledging that wellbeing is essentially social as well as individual, appreciating that wellbeing is experienced in relation to contexts and surroundings” [23]. A public health perspective can also be found in the following definition of health literacy, which entails people’s knowledge, motivation, and competencies to access, understand, appraise, and apply health information in order to make judgments and decisions in everyday life concerning healthcare, disease prevention, and health promotion to maintain or improve quality of life during the life course [27]. To understand this knowledge through studies such as ours is regarded as a precondition for promoting health literacy.

### Data collection

Semi-structured interviews were used to collect empirical data [28]﻿. The researchers visited the locations of local Newcomers groups to establish trust and relations with the groups’ organizers and participants, understand the themes of group meetings on site, and conduct interviews. A purposeful sampling strategy was used to select the organizers and participants in Newcomers. A total of 11 participants (eight men, two women, and one transgender) agreed to participate. The participants were originally from Kurdistan, Ukraine, Iraq, Guinea, Pakistan, Uganda, Nigeria, Russian Republic, Syria, North Macedonia, and Nicaragua. Nine participants migrated to Sweden as LGBTQ refugees due to safety issues regarding their sexuality in their home country, while one migrated due to war, and one migrated due to political persecution. At the time of the study, three participants had been granted asylum, and eight were in the process of seeking asylum. Four of them lived in a big city in Sweden, two lived in a middle-sized city, and five lived in a small-town.

Moreover, interviews with organizers were included to broaden and deepen the range of narratives and gain background information. A total of eight organizers contributed to the study (seven women and one man). The organizers were all Swedish and had a few years of experience with meeting participants in Newcomers or coordinating Newcomers groups (see Table 1).

**Table 1:** RFSL officials organizing Newcomers group

Function within RFSL	Geographical origin
Counselor	Western Sweden
Project manager of Newcomers	Northern Sweden
Counselor	Northern Sweden
Project worker of Newcomers	Northern Sweden
Chair of the local RFSL board	Eastern Sweden
Project manager of Newcomers	Eastern Sweden
National coordinator of Newcomers at RFSL	national level
National coordinator of Newcomers at RFSL Youth	national level

Semi-structured interviews were conducted using specific interview guides for the organizers and the participants. For participants, the interview guide included questions about self-presentation, their migration process and wellbeing, the significance of participating in Newcomers, and their plans for the future. The organizers were asked about basic ideas and developments of Newcomers, their contribution to health and integration, and experiences regarding participants’ developments in the future.

The interviews lasted approximately 30–90 min. All but two interviews were recorded and transcribed verbatim. Two interviews were not recorded due to participants’ apprehensions of prosecution and traumatic experiences with such recordings in their home countries. These interviews were only documented in writing. Most interviews were conducted at the local RFSL premises, which was meant to create a feeling of safety, especially for the participants [28]. The other three interviews were conducted on the phone for practical reasons. However, the researchers and the study participants had met in person before the actual phone interviews, thus establishing a social relation. Establishing such a relationship is crucial for the RFSL organizers as they actively engage in framing safe spaces. The relationship is also crucial for the participants as their decision to participate in the study depended on feeling secure.

Three of the interviews with participants were conducted in Swedish, six were in English, and two were done with an authorized interpreter. Interviews with organizers were conducted in Swedish, including six conducted on site and three on the phone. Information about the study was presented to participants and organizers orally and in writing (in Swedish and English). Moreover, written informed consent was obtained from both the participants and organizers of Newcomers. In addition, the potential mental stress of the study participants was handled by offering counseling services from registered and context-aware counselors working at RFSL. This offer was introduced when informing participants about the study and its conditions before the interviews commenced. The study was approved by the Swedish Ethics Committee (Dnr 2018/734).

### Data analysis

The interview transcription formed a basis for a qualitative content analysis (QCA) [29, 30] with focus on understandings of health. In line with previous arguments [29, 31], we used QCA to focus on the latent content as it was less obvious and demanded interpretation, which matched our research aim regarding the participants’ implicit understandings of health. Interviews with participants and organizers were considered as one empirical source, and the analysis was conducted with all of the material in mind.

To support the aim of the study and present an overview of the interviews, the transcribed data were first read individually by the two first authors who did the analytic groundwork. After the first naïve reading, interpretations were discussed to attain a shared initial understanding of the data. The next step was identifying and condensing meaning units [30], which served as a basis for content condensation and abstraction through coding. Since we focused on understandings of health regarding the wellbeing and ill-being spectrum, tensions in these understandings were emphasized.

The next step was categorizing different codes, resulting in three distinct categories, which were still at a manifest level of abstraction. The final step was creating an overarching theme at the latent level based on the three categories. The analysis process was nonlinear and shifted back and forth between the parts and the entirety of the data during the entire process. Codes, categories, and the theme were discussed among all four authors to ensure trustworthiness, while the two first authors integrated the results of these discussions in the analysis and completed it by engaging in a constant dialogue exchange.

## Results

The data reveal three interconnected, interdependent categories: belonging versus alienation, security and safety versus insecurity, and recognition versus denial, in which tensions occur between wellbeing and ill-being. The three categories contribute to an overarching theme of health as framed freedom – i.e., freedom framed by requirements and environments of society. The categories are described and illustrated below using quotes that are characteristic of each category and indicate the interviews from which the quotes are retrieved (e.g., I-1 for interview 1).

### Belonging versus alienation

Belonging was connected to feelings and practices of human closeness, inclusion, and being responded to positively, with responses ranging from being seen at all (e.g., by answering e-mails, as mentioned by I-7) to recognizing people’s needs, as one of the participants stated: “we [the participants] all have different problems, but they [the organizers] try as much as they can to make us comfortable” (I-5). Belonging was also realized by appreciating people and generate energy, courage, and joy, as one participant described: “We have a lot of fun being here [at RFSL Newcomers]” (I-8). Belonging was elicited when existing members in a social context became involved with the newcomers, supported them, and provided an open, welcoming atmosphere. One of the participants (I-2) refers to this experience when he spoke of RFSL Newcomers, saying, “It felt like we got a new family.”

The participants often generated images of wishing for belonging by describing adverse experiences of alienation in terms of conditioned inclusion, open exclusion, threat, or nonrecognition in different public and private social contexts.

I told my mom, “Mom, I feel like a girl.” But I felt really, really bad, so I went to the hospital (…) They did not have time to talk to me much (…) I have lived in the hospital, like for a while, like a month, and I have received a lot of tablets, and testosterone, and stuffs to be a man. (…) it was actually very difficult to experience [time] in the hospital, that I am not sick (…) why I am here, because I feel, I like guys, or, and I tried to take my life (I-1).

Contexts for adverse experiences span across nations and different group settings, relatives, friends, and the Swedish Migration Agency (SMA). According to the experiences of participants, being assigned an identity category that is fitting with one’s understanding of oneself by others functions as a condition of belonging and feeling well in all contexts. Overall, belonging exists in tension with alienation and is characterized by experiences of fitting in, being unconditionally accepted, or feeling activated, in contrast to feeling like a misfit, being merely conditionally accepted, or feeling passivized when alienated.

Belonging was also highlighted by the organizers as one important condition for Newcomers. Participation inherently would lead to a sense of belonging:

… a lot of people feel very lonely… and a lot of people think it’s just them… “it’s just me in the whole world that is like this”… and many become friends and don’t have to feel so lonely and to understand that others/that there are others who have fled for the same reason; there are others who have also been imprisoned or have been subjected to murder attempts or been ostracized by their families, that you are not alone, and it is clear that it does something when you see it. (counselor, Western Sweden)

Like the participants, even organizers acknowledged a gap, where LGBTQ newcomers risked being alienated in the Swedish queer-community spaces and in their home-cultural setting, which eventually led to the formation of Newcomers:

Some people were rather vulnerable and could not be in Sweden’s queer room – in other words, LGBTQ rooms – because they were exposed to racism and alienation, as well as cultural differences. And they couldn’t go to, for instance, the Somali association and hang there because they were LGBTQ, and that sexual orientation was not accepted. So that’s why we needed some kind of space for this target group. (National Coordinator)

Belonging was thus risky and ambivalent. On the other hand, it might work as a social anchor and serve as a basis for other beneficial inputs to enhance wellbeing as well.

### Recognition versus denial

Recognition was characterized by feelings of appreciative acknowledgment, acceptance, respect, and a mutual quality, as one of the participants puts it: “I… respect people and get respect back. When I came to Sweden, then it wasn’t a shock among the Swedish people (…) I opened up my sexuality (…) from the beginning. They helped, and there was no hatred or anything, but they accepted me” (I-11).

Recognition was also received from other people and achieved for others and oneself, both as a person and for (representations of) personal identity. Thus, a legal, social, and self-referential dimension is part of the recognition, which assumes an important role in personal identity building and socially induced normalization within the RFSL context. One of them exemplified the individual dimension by claiming, “if you’re a self-seeker, [RFSL] is just [the] place to be” (I-8). Another illustrated the social dimension by stating, “By meeting people like me, I feel more comfortable and I feel comfortable when I open up my homosexuality status” (I-3), showing mutual acknowledgment in the Newcomers group and its empowering effect.

The legal dimension is important during the asylum process, especially during the interview at the SMA. Here, the tension between recognition and denial became visible. Most participants who were still seeking asylum described a lack of recognition from the administration regarding their story and the way they presented their lives. As this denial could lead to not being granted access to Swedish society, the construction of denial appeared as a feared antipode to recognition. One of the participants gave an example of this as a lack of acknowledgment and the helplessness it produced:

“Migration” knows the story (…) in the interview, you said what happened, but they wanted to know the details when they raped you, what they were saying when they were raping you. It’s hard, I mean… they raped me… I told them [the migration officers] they hit me, everything they did to me, a bit like more and more details, it’s not so easy. And when they ask, it’s so hard you know, and they just say, “We need a strong story, like more” I say, “what more?,” they broke my arm, they had me one month, they raped me… what more can I say? (I-9)

Here, the connection to questions of power becomes apparent as recognition cannot be performed by everybody, only those “in charge.” Moreover, acknowledgement of belonging to a category may be empowering or depowering. The legal, social, and self-referential dimensions of recognition were supported by organizers:

We also have our counselor, who often has contact with those who are members of the Newcomers group too… because many need to get support on various issues. And partly because you think it is difficult in the asylum process, but also because of past experiences, you may need to talk about your identity to figure it out, and you may not have had words to express who you are before... we also have national lawyers within RFSL who can advise those who are in asylum processes. They do not take cases themselves, but they can help with consultation to the lawyer who has the case. (organizer, Northern Sweden).

In all cases, a person’s categorial belonging was investigated and determined. This indicates a close link between belonging and recognition. Thus, recognition is a result of determining who someone “really” is, as the organizer points out. Identity formation appears to be understood in two ways: the notion of a “real” identity reveals a basic essentialist assumption, whereas the need to negotiate one’s identity within a new linguistic space of possibilities highlights a processual and contextual character of identity formation. Both are represented in I-8’s aforementioned self-seeking: whereas the seeking represents the process, the self stands for the essential result expected to surface during the seeking process.

The three processes of legal, social, and self-related recognition were described as co-occurring and lead to very different results. When these recognition dimensions work in harmony, wellbeing conditions are favorable. Whereas the SMA was described as a threat to that alignment and a representative of denial, RFSL presented themselves as non-judgmental as they were transparent about not being able to determine someone’s sexual orientation and gender identity. This was elucidated by one of the organizers: “We say that we cannot write a certificate that you are an LGBTQ person; I cannot certify it; only you have that identity, and you are the owner of that identity. However, we can certify that you are a member; we can certify that you have been here, and that is what we can do” (organizer, Northern Sweden).

### Security and safety versus insecurity

The category of security and safety was characterized as being free from threats with regard to either an existential or a spatial dimension and included participants’ commitment to cope with their vulnerability. Being safe and secure is critical to enhanced wellbeing. Moreover, being secure and safe from spatial threats (i.e., feeling safe in a location) is the primary condition for feeling included in a social milieu and exploring one’s identity. As presented by one of the participants: “Yeah, first of all… the main thing is to be safe” (I-3). The participants did not feel safe and secure in unfavorable social circumstances, pointing to a counterpoint of insecurity that threatens wellbeing.

Regarding the existential dimension of security and safety, the antipode of severe insecurity is described as a direct threat to physical life and mental health. Insecurity became apparent in participants' narration about their home country, family, and flight. For example, one of the them (I-4) was still afraid of his father, who joined him on his flight from a war-afflicted country to Sweden. The fear was associated with the force that his father may use to make him behave according to what his father considered manly.

Immigrating to Sweden was often presented as reaching safety: “When I sat down on the flight, I felt my suffering was over” (I-11). That feeling was at times questioned by experiences of insecurity in the Swedish refugee camps, in which participants met people with the same derogative view of being LGBTQ as in their home countries. Here, the spatial and existential dimensions of safety, security, and insecurity overlap, providing a safe haven for personal existence – or precisely the opposite. One of the participants describes: “Actually, I don’t feel good at the camp, we are 20 people… from [2 countries], and they are Muslims, and I feel scared because I’m gay, and they don’t like homosexuals, they don’t like tattoos, and in some sense, I’m afraid (…) I don’t feel comfortable there” (I-10). Thus, both the LGBTQ refugees’ homelands and the refugee camps represented a potential danger as discursively constructed spaces of insecurity.

Moreover, the availability and application of therapeutic and medical resources are critical to making LGBTQ refugees feel safe and secure since many must deal with trauma or illness. Here, some participants like I-4 described how they were helped by RFSL counsel in dealing with traumatic experiences.

In addition, the tension between security and insecurity was highlighted in the spatio-cultural dimension, involving the task of occupying and orientating oneself with norms and values in certain milieus to feel safe. The goal was to feel secure and familiar with the required expectation “not to do stupid things that Swedes don’t accept” (I-2) in these living conditions. To feel safe and secure, it was expected of them to navigate Swedish society and become culturally and socially literate. One of the roles of the organizers was to facilitate contact with other agents in society: “It is really to get them out at workplaces or schools… to learn Swedish, meet Swedes…. Now they have met us a lot, but we are not enough, they need to meet even more Swedish friends” (chairman of the local RFSL board, Eastern Sweden).

Organizers took up roles as intermediaries, which matched the sentinel-like gatekeeper role they assumed during the data collection. Thus, they acted as buffers against society, and they are often considered as such by participants. For instance, regarding Swedish behavior and information about how Sweden functions, one of the participants explains: “(…) we have daily [RFSL-organized] training, where we can learn how Swedish society functions – how important it is to follow the law in Sweden… what you can do, and where you can ask for help” (I-8). Judging from these different ways of becoming safe and secure, “safety and security versus insecurity” are elements of wellbeing that represent not a status but a process that may be challenged in social situations.

### Framed freedom

**Fig. 1:**
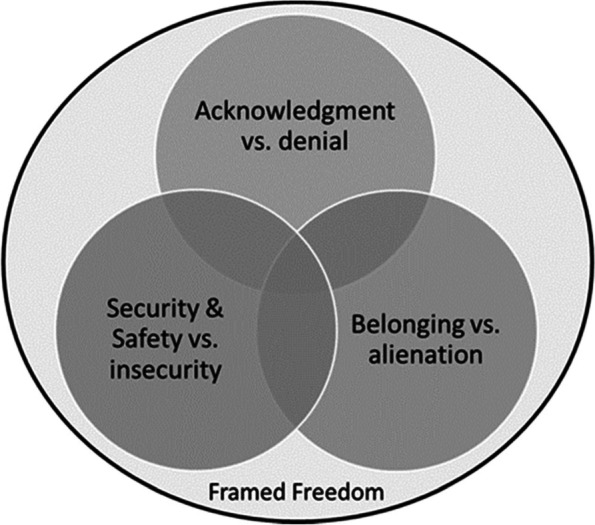
The interrelation of different understandings of health

The three categories of belonging versus alienation, recognition versus denial, and security and safety versus insecurity are interrelated and social in nature. Their interrelation is mutually enhancing and results in increasing freedom as the overarching theme that comprises the ability to live ideally in a completely self-determined and self-realized manner after finishing a process of recognition by the LGBTQ refugees themselves and by the Swedish authorities. Recognition allows individuals to belong to the nation of Sweden and have a feeling of being safe and secure within its cultural frame. Hence, the theme focuses on framed freedom. One person elaborates that being free is synonymous with being safe, recognized, and belonging, which illustrates how the theme is built on the three categories: “I feel free here; I feel that I should not hide my identity; I am not ashamed that I am the way I am, and I feel safe doing what I want” (I-10). Figure 1 shows this interactive relation between the categories of belonging versus alienation, recognition versus denial, and security and safety versus insecurity for the emergence of framed freedom.

Similar to these categories, framed freedom also includes a process-related component. In another part of the interview, one participant describes how he has little hope of being allowed to reside in Sweden permanently. This complements the potential to be free here and now with the notion of a change over time, eventually ending in a state in which a permanent resident permit serves as a final guarantor of safety and security. Thus, the freedom is also framed by demands for recognition and belonging, which are often represented by the “needle’s eye” of the migration interview, and the prospect of freedom exists in tension with a feared lack of prospects:

I have a lot to think about, so I can’t think about the future, and I can’t think of what my future will be like (...) I’m terrified. I’m afraid they’ll send me back to [my home country]. (I-10)

Even when participants receive permanent permission to reside in Sweden, the prerequisites for freedom are still framed by circumstances connected to being LGBTQ: “some may feel that… outside Newcomers, you can’t be open to anyone, at least not someone who is from the same country, because you are afraid it [information] will reach your parents or others” (counselor, Western Sweden). To sum up, freedom is framed by the results of the identity process and by the way in which LGBTQ people are received in society and blend in culturally.

Concerning freedom, the role of RFSL is changing from being a guardian-supporter to being equal. Often, participants position RFSL Newcomers as an organization to which they owe some debt that they must pay back:

(...) the [RFSL Newcomers group] has been very helpful (...) I know how they helped me and checked on me, I know (...) I should also help other new people coming in, you understand? So, I should (?1) help them to help others. So, they give it to us, we should also give it to (1?). (I-6)

In such cases, they adopt the role of the organization without questioning. It may be assumed that paying back the “debt” is the last step of becoming truly free.

Other participants in Newcomers talked about committing themselves to the political fight – either for LGBTQ or other people’s rights – and might intend to work for change within RFSL or on a broader societal level:

The struggle, it’s not just saying at the Pride Festival: “okay, we have Newcomers, and we have the rainbow law; we are RFSL; we take care of them, we fight for rights for LGBTQ people and so on, but it’s not just a way to speak; so it’s not the words you say; you have to do something, that’s what I’m thinking. (I-1)

## Discussion

The aim of this study was to reconstruct LGBTQ refugees’ understandings of health. Our data reveal three interconnected, interdependent categories that contribute to one overarching health theme. Belonging versus alienation, security and safety versus insecurity, and recognition versus denial must be navigated to achieve freedom framed by societal requirements and environments. Understandings of health and the identity of a healthy subject are constructed and developed in these tensions within the frame of the RFSL Newcomers groups, which has theoretical and practical implications.

### Theoretical implications

The LGBTQ refugees understood and constructed health in terms of variations of belonging, recognition, and security/safety, which thus appear as “elements” of how health is understood. In other words, when understandings of health are reconstructed from general narratives on migration and framed as congruent to wellbeing, as in this case, belonging, recognition, and security/safety assume the role of *conceptual elements* of health. As all of these categories are relational and have existential properties, the resulting theme of “health as framed freedom” transcends the role that typically is assigned to freedom regarding health. Usually, freedom is understood from an individualistic, neoliberal point of view, which is characterized by voluntarily demonstrating individual responsibility, prudence, health consciousness, health commitment and productivity regarding the construction of one’s healthiness. In doing so, such a person proves to be a good citizen in relation to health, a so-called good bio-citizen [32–34]. In following this rationale of our participating LGBTQ refugees, our results challenge this hitherto established scientific understanding [35–38], which identifies belonging, recognition, and safety/security as *causal social determinants* of health for migrants (with health being conceptualized differently). In other words, so far, belonging, recognition, and safety/security have been regarded as conditions leading to health instead of components of health, describing the experience of health itself.

Moreover, the results show that there is an interdependence between the construction of individual self-defined identity and the construction of health. These processes are framed by the variety of health discourses that is present in interactions within certain social contexts [39]. Forming an individual’s identity under these circumstances comprises the determination and realization of oneself as “who I am,” which is a task that has been identified as challenging for LGBTQ refugees [18] and calls upon essentialist and process-oriented interactionist concepts of identity [40]. In our study, the identity formation of LGBTQ refugees is described as a liberation from insecurity, alienation, and denial that changes over time and interacts with health. Both health and identity are mutually reinforced in a positive way when belonging, security/safety, and recognition are experienced.

Beneficial belonging is understood as feelings of closeness, inclusion, and active support that generate joy, energy, and courage, crucial for the LGBTQ migrant experience [41, 42]. Recognition is characterized by acknowledgment, acceptance, and respect, experienced socially, legally, and self-focused. This description fits Honneth’s theory [43] of intersubjective recognition, emphasizing emotional concern, respect, and a positive evaluation of one’s capacities and traits as preconditions for positive self-relation enabling the development of personal identity and a good life in freedom [43]. Hence, recognition is proving to be a “vital human need” [44, p. 26]. Our results confirm notions of interrelatedness between health and multidimensional identity work concerning cultural, sexual, or gender identity [45], which becomes necessary when in a new society [46]. This has a bearing on participants’ experiences and thoughts about the future [47].

### Practical implications

The following practical implications may help to develop public health and promote health literacy among refugees [23, 26, 27, 48]. By switching from self-defined individual identity to other-defined social/categorical identity [40], issues of power and its practical implications are addressed by discussing minority and majority positions, which provide orientations for health and identity formation. It is here that the roles of stakeholders like RFSL and the SMA come into play. Two main positions are interrelated in this study. First, the LGBTQ refugees represent a minority group in society. Typically, an LGBTQ refugee is in a potentially anomic situation; i.e., norm-lacking, in-between ethnic cultures (as “sexual outsiders”) and sexually connoted subcultures (as “ethnic outsiders”), yet with a distinct will to preserve an embodied self being at risk [49]. Second, RFSL as an organization represents the perspective of the cultural majority (concerning ethnicity) and the subcultural minority (concerning sexuality). Thus, RFSL’s in-house discourse is regarded as providing relevant orientation concerning the process of identity formation. The SMA adds to both ethnic and sexual-majority discourses, functioning as an evaluator of a trustworthy LGBTQ presentation, an evaluator that grants resident permits as a sign of approving LGBTQ refugees’ trustworthy self-presentation [10, 50].

As LGBTQ representatives, RFSL personnel suggest and exhibit an orientation for identity formation that must be marked by Western concepts of “being LGBTQ.” These concepts comprise several outspoken self-designations for LGBTQ persons, which deliver a frame for recognition and belonging as “one of us.” Moreover, granted that cultural literacy information (“how Swedish society functions” as I-8 puts it) is an important part of Newcomer meetings in many visited groups, RFSL provides an orientation that questions the participant’s existing ethnic concepts of conduct and may replace it with an ethnic practice that is suitable in Swedish culture. For example, a strong orientation towards feeling safe and secure in Swedish society is promoted based on a paramount concept of security and safety at RFSL, which is enacted by RFSL organizers when operating as guardians and gatekeepers with regard to regulating access to participants in Newcomers.

RFSL as a civil societal organization acts under different circumstances compared to the state. RFSL receives funding from the state to assist in the reception of refugees. RFSL assists LGBTQ refugees to assure their rights in relation to the state and obtain a residence permit in Sweden. Therefore, as part of civil society, RFSL has more discretion (freedom of action) than the state. This allows RFSL to provide different, more pragmatic, and close support in terms of safety, belonging, acknowledgment, and practical support. Thus, RFSL may present a free haven and respite during a time of passage, in which the state still has not stepped in to give support at all, but also as an addition after state involvement begins. Herein lies a chance for a collaboration and a mutual completion of what the state and RFSL as a civil society organization may be able to provide for LGBTQ refugees, with regard to both acculturation and health (literacy).

Going back to orientations, although the orientations mentioned are based on the needs of LGBTQ-refugees, their provision simultaneously constructs normative objectives for “feeling at home” regarding identity, integration, and health as a home-like being in the world [49, 51]. Even SMA uses ethnocentric norms regarding “being LGBTQ” to guide their asylum decisions [10]. Normative objectives must function as directing guidelines rather than suggesting guidelines for LGBTQ refugees. The objectives indicate assimilation (or withdrawal) as a way to gain security, belonging, recognition, and wellbeing as a framed freedom due to LGBTQ refugees’ dependable position as a minority. Our results are consistent with existing research [17], indicating that many of the participants already adhere to these orientations, thereby constructing themselves as “good migrants” and “good LGBTQ people.” However, some participants (with permanent resident permits) question expectations regarding integration and LGBTQ, which hint towards a change of perspective and position connected to the granted asylum.

In each case, being a “good (LGBTQ) migrant” implies inhabiting a limited subcultural and ethicized cultural space that allows one to express one’s identity while feeling increasingly familiar within this space, which may facilitate cultural relaxation. Hence, a circular process of identity formation emerges, in which different normative notions of “home” (concerning identity, culture, and health) are increasingly been aligned. This is consistent with previous research in which a participant of a health information program including Swedish civic introduction course illustrated this connection by expressing that “to take care of one’s health is a start of our integration in Sweden” [35, p. 7].

However, there is still a potential inbuilt contradiction: adhering to the normative objections may promote the relational and existential health described by our participants, which challenges the normative expectations inherent to the hegemonic biomedical discourse with its individualized healthistic bio-citizens [33, 52]. Hence, a follower of the mentioned orientations might turn out to be both a “good migrant” and a “bad bio-citizen.” That is, the intervention in question may not clearly opt for empowerment as realizing oneself as a responsible welfare consumer [53], as well as the promotion of individual bio-citizenship and a biomedical understanding of health, as discussed by Al-Adhami et al. [35]. In this case, the practical challenge is hence to decide which understanding of health one wants to choose as a foundation for one’s intervention as the biomedical one may result in “good bio-citizens,” whereas the relational and existential one (endorsed by our participants) may lead to enhanced experiences of subjective wellbeing.

### Limitations and future research

This study is limited by the selection of participants from the Newcomers groups. It is evident that LGBTQ refugees feared being detected, and they were vulnerable to oppression and violence, which prevented some of them from participating and led to a limited number of study participants. Moreover, many of the (asylum-seeking) participants were in a position of dependency on RFSL during the interview. This position could have led them to present overly positive presentations of RFSL Newcomers. However, this is not deemed to be a limitation of our study as it does not question understandings of belonging, security/safety, and recognition within health as a framed freedom, judging from the contributions of participants with a permanent resident permit.

This study does not explicitly explore LGBTQ refugees’ health understandings through health-related definitions, practices, experiences, and so on. Instead, the study explores health’s implicit relevancy and its role in LGBTQ refugees’ migration process. Thus, analytical attention must be directed towards health understandings. As we applied a broad and open approach to health, we regard our procedure as being able to meet the aim of this study. Not approaching the topic explicitly is actually deemed rather beneficial as we reconstruct how health as wellbeing is understood in everyday situations. We suggest that comparing explicit and implicit understandings of health could be an interesting task in future research.

Using interpreters in some interviews is another presumable limitation as loss of information could have occurred. Moreover, the number of interpreters is limited, and they may be integrated into the respective ethnic minority culture, which may lead to a power imbalance and enhance feelings of vulnerability on the part of the LGBTQ person being interviewed [54, 55]. We attempted to minimize these potential limitations by using licensed interpreters and conducting interviews with interpreters on the telephone to avoid face-to-face recognition. However, if participants speak in their preferred language, this could enhance the quality of their stories and the data collected [55].

Following Mangrio et al. [54], we suggest that the selection process was a strength of the study, given the wide array of different national backgrounds of participants and different local RFSL branches that participated. The Newcomers groups are from different geographical backgrounds. In addition, the researchers visited Newcomers’ group activities and established relations with participants, which enhanced relationships and trust.

Future research could focus on the relation between means of integration [56] and the development of an opinion about a new “home” that may be reconfigured and critically questioned. Therefore, it is valuable to study participants in Newcomers (and former participants) with or without a permanent residence permit using a longitudinal approach. Moreover, we suggest comparing experiences between (LGBTQ) migrants who have achieved permanent residency and those not yet granted asylum, as we already have clues about their differing points of view, potentials, and needs in this small sample.

## Conclusion

From a public health perspective, this study provides useful knowledge about subjective understandings of health expressed by LGBTQ refugees (supported by organizers’ views) who participated in a Swedish health-related integration intervention, RFSL Newcomers, and the interplay between identity and health. The results could potentially be useful for increasing health literacy, as described in earlier research on refugees. Belonging, recognition, and security/safety are deemed conceptually relevant to experiencing health as framed freedom, which is simultaneously challenged by alienation, denial, and insecurity. This freedom is conditioned by societal and practical circumstances and requirements that give directions to health and identity, which may or may not allow access to the Newcomers group and the Swedish society.

RFSL Newcomers appears to play a significant role as a representative model of the cultural majority and provider of directions, thereby facilitating a healthy resettlement process for LGBTQ refugees in Sweden. Determining what understanding of health to use as a basis for such interventions (and thus “healthy” resettlement) is a task that needs to be consciously addressed by interventions organizers to support biomedically based bio-citizenship or create conditions for the promotion of a more relational, existential wellbeing. Generally, organizers of interventions may enhance the effectiveness of their interventions when relational, existential, and biomedical understandings of health are all incorporated. 

## Data Availability

The datasets used or analyzed during the current study are available from the corresponding author on reasonable request. To protect the participants’ identities and due to the type of consent we received from the participants, the full interview data of this study (transcripts and audio files) will not be made available to the public.

## Abbreviations

LGBTQ: Lesbian, gay, bisexual, transgender, or queer
RFSL: Swedish Federation for LGBTQ Rights
SMA: Swedish Migration Agency

## References

[1] UNHCR. Figures at glance. 2020. http://www.unhcr.org/figures-at-a-glance.html. Accessed 13 Feb 2020.

[2] Eurostat. Eurostat regional yearbook. 2018 ed. Luxembourg: Publications Office of the European Union; 2018.

[3] Agergaard S (2011). Development and Appropriation of an Integration Policy for Sport: how Danish Sports Clubs have Become Arenas for Ethnic Integration. Int J Sport Policy Politics.

[4] Ambrosini M, Van der Leun J. Introduction to the Special Issue: Implementing Human Rights: Civil Society and Migration Policies. J Immigr Refug Stud 2015; doi:10.1080/15562948.2015.1017632.

[5] Fredriksson I, Geidne S, Eriksson C (2018). Leisure-time youth centers as health-promoting settings: experiences from multicultural neighborhoods in Sweden. Scand J Public Health.

[6] Field J. Social capital and lifelong learning. Bristol: Policy; 2005.

[7] Portes A, Rumbaut RG (2001). Legacies: the story of the immigrant second generation.

[8] Zhou M, Kim SS (2006). Community Forces, Social Capital, and Educational Achievement: The Case of Supplementary Education in the Chinese and Korean Immigrant Communities. Harv Educ Rev.

[9] RFSL. Briefly about RFSL. 2020a. https://www.rfsl.se/en/about-us/kort-om-rfsl. Accessed 16 Jan 2020.

[10] Gröndahl A. Reasons for refusal in LGBTQI cases. Stockholm: RFSL; 2020.

[11] Logie C, Lacombe-Duncan A, Lee-Foon N, Ryan S, Ramsay H. “It’s for us – newcomers, LGBTQ persons, and HIV-positive persons. You feel free to be”: a qualitative study exploring social support group participation among African and Caribbean lesbian, gay, bisexual and transgender newcomers and refugees in Toronto, Canada. BMC Int Health Hum Rights. 2016. 10.1186/s12914-016-0092-0 10.1186/s12914-016-0092-0 PMC4930565 27369374

[12] Fox SD, Griffin RH, Pachankis JE (2020). Minority stress, social integration, and the mental health needs of LGBTQ asylum seekers in North America. Soc Sci Med.

[13] Roth N, Boström G, Nykvist K. Hälsa på lika villkor? Hälsa och livsvillkor bland HBT-personer. [Health on equal terms? Health and living conditions among LGBT-people]. Folkhälsomyndigheten. 2006. https://www.folkhalsomyndigheten.se/contentassets/b3aab8ab230c4c798a95695e87f93882/hbt_web.pdf. Accessed 13 Feb 2020.

[14] Eliason MJ, Chinn PL. LGBTQ cultures. What health care professionals need to know about sexual and gender diversity. 3rd ed. Philadelphia: Wolters Kluwer; 2018.

[15] Murray DAB (2014). The Challenge of Home for Sexual Orientation and Gendered Identity Refugees in Toronto. J Can Stud.

[16] Viruell-Fuentes EA, Miranda PY, Abdulrahim S (2012). More than culture: Structural racism, intersectionality theory, and immigrant health. Soc Sci Med.

[17] Pachankis JE, Hatzenbuehler ML, Berg RC, Fernández-Dávila P, Mirandola M, Marcus U, Weatherburn P, Schmidt AJ (2017). Anti-LGBT and Anti-immigrant Structural Stigma: An Intersectional Analysis of Sexual Minority Men’s HIV Risk When Migrating to or Within Europe. J Acquir Immune Defic Syndr.

[18] Fournier C, Hamelin Brabant L, Dupéré S, Chamberland L (2018). Lesbian and Gay Immigrants' Post-Migration Experiences: An Integrative Literature Review. J Immigr Refug Stud.

[19] EJ Alessi S Kahn B Greenfield L Woolner D Manning 2020 A Qualitative Exploration of the Integration Experiences of LGBTQ Refugees Who Fled from the Middle East, North Africa, and Central and South Asia to Austria and the Netherlands Sex Res Soc Policy 2020 10.1007/s13178-018-0364-7

[20] RFSL. Newcomers. 2020b. https://www.rfsl.se/en/organisation/asylum/asyl. Accessed 26 March 2020.

[21] Kickbusch I, Kickbusch I, Potvin L, Balbo L, Pelikan JM, Abel T (2007). Health Governance: The Health Society. McQueen DV.

[22] Pelters P (2021). Right by your side? – the relational scope of health and wellbeing as congruence, complement and coincidence. Int J Qual Stud Health Well-being.

[23] Dooris M, Farrier A, Froggett L (2018). Wellbeing: The challenge of ‘operationalising’ a holistic concept within a reductionist public health programme. Perspect Public Health.

[24] Pons-Vigués M, Berenguera A, Coma-Auli N, Pombo-Ramos H, March S, Asensio-Martínez A, Moreno-Peral P, Mora-Simón S, Martínez-Andrés M, Pujol- RE (2017). Health-care users, key community informants and primary health care workers’ views on health, health promotion, health assets and deficits: Qualitative study in seven Spanish regions. Int J Equity Health.

[25] Blaxter M (2010). Health.

[26] WHO. Report on the health of refugees and migrants in the WHO European Region. No public health without refugee and migrant health. 2018. https://apps.who.int/iris/bitstream/handle/10665/311347/9789289053846-eng.pdf?sequence=1&isAllowed=y. Accessed 25 April 2022.

[27] Sørensen K, Van den Broucke S, Fullam J, Doyle G, Pelikan J, Slonska Z (2012). Health literacy and public health: a systematic review and integration of definitions and models. BMC Public Health.

[28] Merriam SB. Qualitative Research. A guide to design and implementation. San Francisco: Jossey Bass; 2009.

[29] Schreier M (2012). Qualitative content analysis in practice.

[30] Graneheim UH, Lundman B (2004). Qualitative content analysis in nursing research: concepts, procedures and measures to achieve trustworthiness. Nurse Educ Today.

[31] Vaismoradi M, Turunen H, Bondas T (2014). Content analysis and thematic analysis: Implications for conducting a qualitative descriptive study. Nurs Health Sci.

[32] M Jong De A Collins S Plüg 2019 “To be healthy to me is to be free”: how discourses of freedom are used to construct healthiness among young South African adults Int J Qual Stud Health Well-being 2019 10.1080/17482631.2019.1603518 10.1080/17482631.2019.1603518 PMC6493219 31033428

[33] Halse C, Harwood V (2009). Bio-Citizenship: Virtue discourse and the birth of the bio-citizen. Wright J.

[34] Rose N (1999). Powers of freedom: reframing political thought.

[35] Al-Adhami M, Hjelm K, Wångdahl J, Larsson EC (2021). “This course is like a compass to us” – a qualitative study on newly settled migrants’ perceptions of civic and health orientation in Sweden. BMC Public Health.

[36] Caxaj CS, Gill NK (2017). Belonging and Mental Wellbeing among a Rural Indian-Canadian Diaspora: Navigating Tensions in "finding a Space of Our Own". Qual Health Res.

[37] L Mwanri L Anderson K Gatwiri 2021 Telling Our Stories: Resilience during Resettlement for African Skilled Migrants in Australia Int J Environ Res Public Health 2021. 10.3390/ijerph18083954 10.3390/ijerph18083954 PMC8069641 33918671

[38] Ziersch A, Miller E, Baak M, Mwanri L (2020). Integration and social determinants of health and wellbeing for people from refugee backgrounds resettled in a rural town in South Australia: a qualitative study. BMC Public Health.

[39] James A, Hockey J (2007). Embodying health identities.

[40] Westin, Charles. Citizenship and identity. In: A. Kondo A, Westin, C, editors. New Concepts of Citizenship. Residential/Regional Citizenship and Dual Nationality/Identity. Stockholm University: CEIFO; 2003, p. 171–204.

[41] Lee E, Jin O, Brotman S (2011). Identity, Refugeeness, Belonging: Experiences of Sexual Minority Refugees in Canada. Can Rev Sociol.

[42] Asante G (2018). "Where is home?" Negotiating Comm(unity) and Un/Belonging Among Queer African Migrants on Facebook. Borderlands.

[43] Honneth A (1995). The Struggle for Recognition.

[44] Taylor C, Gutmann A (1992). The Politics of Recognition. Multiculturalism: Examining the Politics of Recognition.

[45] Fuks N, Smith NG, Peláez S, De Stefano J, Brown TL (2018). Acculturation Experiences Among Lesbian, Gay, Bisexual, and Transgender Immigrants in Canada. Couns Psychol.

[46] Walseth K (2006). Sport and Belonging. Int Rev Sociol Sport.

[47] Kostenius C, Hertting K, Pelters P, Lindgren EC (2021). From hell to heaven? - lived experiences of LGBTQ migrants in relation to health and their thoughts about the future. Cult Health Sex.

[48] J Wångdahl P Lytsy L Mårtensson R Westerling 2014 Health literacy among refugees in Sweden - a cross-sectional study BMC Public Health org/10.1186/1471-2458-14-1030 10.1186/1471-2458-14-1030 PMC4195944 25278109

[49] Wimark T (2021). Homemaking and perpetual liminality among queer refugees. Soc Cult Geogr.

[50] Dhoest A (2019). Learning to be gay: LGBTQ forced migrant identities and narratives in Belgium. J Ethn Migr Stud.

[51] Svenaeus F (1999). The hermeneutics of medicine and the phenomenology of health: steps towards a philosophy of medical practice.

[52] Crawford R (2006). Health as a meaningful social practice. Health.

[53] Askheim OP (2003). Empowerment as guidance for professional social work: An act of balancing on a slack rope. Eur J Soc Work.

[54] Mangrio E, Carlson E, Zdravkovic S (2020). Newly arrived refugee parents in Sweden and their experience of the resettlement process: A qualitative study. Scand J Public Health.

[55] K Ingvarsdotter S Johnsdotter M Ostman 2012 Lost in interpretation: the use of interpreters in research on mental ill health Int J Soc Psychiatry 2012 10.1177/0020764010382693 10.1177/0020764010382693 20833705

[56] Ager A, Strang A (2008). Understanding Integration: A Conceptual Framework. J Refug Stud.

